# In Vitro Evaluation, Chemical Profiling, and In Silico ADMET Prediction of the Pharmacological Activities of *Artemisia absinthium* Root Extract

**DOI:** 10.3390/ph17121646

**Published:** 2024-12-07

**Authors:** Asma N. Alsaleh, Ibrahim M. Aziz, Reem M. Aljowaie, Rawan M. Alshalan, Noorah A. Alkubaisi, Mourad A. M. Aboul-Soud

**Affiliations:** 1 Department of Botany and Microbiology, College of Science, King Saud University, Riyadh 11451, Saudi Arabia; asmalsaleh@ksu.edu.sa (A.N.A.); raljowaie@ksu.edu.sa (R.M.A.); ralshalaan@ksu.edu.sa (R.M.A.); nalkubaisi@ksu.edu.sa (N.A.A.); 2 Department of Clinical Laboratory Sciences, College of Applied Medical Sciences, King Saud University, P.O. Box 10219, Riyadh 11433, Saudi Arabia; maboulsoud@ksu.edu.sa

**Keywords:** *Artemisia absinthium* root extract (AARE), natural products, bioactive compounds, bioactive compounds, molecular docking

## Abstract

*Artemisia absinthium* L., is a plant with established pharmacological properties, but the *A. absinthium* root extract (AARE) remains unexplored. The aim of this study was to examine the chemical composition of AARE and assess its biological activity, which included antidiabetic, antibacterial, anticancer, and antioxidant properties. GC-MS was used to analyze the chemical components. The antioxidant activity of the total phenolic and flavonoid content was evaluated. Antibacterial activity and cytotoxic effects were identified. Enzyme inhibition experiments were performed to determine its antidiabetic potential. Molecular docking was utilized to evaluate the potential antioxidant, antibacterial, and anticancer activities of the compounds from AARE using Maestro 11.5 from the Schrödinger suite. AARE exhibited moderate antioxidant activity in DPPH (IC_50_: 172.41 ± 3.15 μg/mL) and ABTS (IC_50_: 378.94 ± 2.18 μg/mL) assays. Cytotoxicity tests on MCF-7 and HepG2 cancer cells demonstrated significant anticancer effects, with IC_50_ values of 150.12 ± 0.74 μg/mL and 137.11 ± 1.33 μg/mL, respectively. Apoptotic studies indicated an upregulation of pro-apoptotic genes (*caspase*-*3*, *8*, *9*, *Bax*) and a downregulation of anti-apoptotic markers (*Bcl-2* and *Bcl-Xl*). AARE also inhibited α-amylase and α-glucosidase, suggesting potential antidiabetic effects, with IC_50_ values of 224.12 ± 1.17 μg/mL and 243.35 ± 1.51 μg/mL. Antibacterial assays revealed strong activity against Gram-positive bacteria. Molecular docking and pharmacokinetic analysis identified promising inhibitory effects of key AARE compounds on NADPH oxidase, *E. coli* Gyrase B, and Topoisomerase IIα, with favorable drug-like properties. These findings suggest AARE’s potential in treating cancer, diabetes, and bacterial infections, warranting further in vivo and clinical studies.

## 1. Introduction

Wormwood, also known as *Artemisia absinthium* (*A. absinthium*), is a herbaceous plant from the Asteraceae family. It was formerly referred to as “the most important master against all exhaustions” in medieval Europe. It is a species that is used as medicine in North America, West Asia, and Europe [1]. This plant is known for its bioactive components, including flavonoids, phenolic acids, terpenoids, and essential oils, which contribute to its antioxidant, anticancer, antibacterial, and antidiabetic properties [2,3]. Despite widespread interest in the plant’s aerial parts, the root extract of *A. absinthium* is understudied, providing an opportunity to research its possible pharmacological effects.

*A. absinthium*’s antioxidant effects are ascribed to its high quantities of phenolic compounds, which scavenge free radicals and reduce oxidative stress, two variables linked to a variety of chronic illnesses, including cancer and diabetes [4]. Previous research has shown that the aerial portions of the plant have high antioxidant activity, implying that the roots may have similar or improved benefits [5]. This antioxidant capability is critical because it can act as a defense mechanism against oxidative damage, which is connected to aging and degenerative disorders [6].

In vitro studies have revealed that *A. absinthium* extracts cause apoptosis in cancer cell lines, underlining the plant’s potential as a natural chemotherapeutic agent [7,8,9]. Furthermore, *A. absinthium* has antibacterial capabilities against a wide range of bacteria and fungi, demonstrating its feasibility as a source of natural antimicrobial agents [3,10,11].

*A. absinthium* has been shown to inhibit key enzymes involved in glucose metabolism, including α-amylase and α-glucosidase, making it an effective antidiabetic [12,13]. These characteristics make *A. absinthium* an attractive option for treating postprandial hyperglycemia and maybe aiding in diabetes management.

Molecular docking is a frequently used computer approach for predicting the interactions between bioactive chemicals and target proteins [14]. In the context of plant extracts, this approach aids in the identification of possible bioactive chemicals that may contribute to the plant’s therapeutic benefits by assessing their interaction with particular molecular targets implicated in various illnesses [15]. Molecular docking not only helps researchers understand the method of action of these molecules, but it also aids in virtual screening, allowing them to prioritize compounds for future experimental confirmation [16]. Molecular docking can identify compounds in *A. absinthium* root extract (AARE) that bind strongly to key therapeutic targets, including enzymes involved in oxidative stress (e.g., NADPH oxidase), cancer proliferation (e.g., Topoisomerase IIα), bacterial survival (e.g., DNA Gyrase B), and diabetes (e.g., α-glucosidase) [14]. Researchers can anticipate the possible pharmacological activity of specific chemicals in the extract by analyzing their binding scores, hydrogen bonding, and hydrophobic interactions [17,18].

Pharmacokinetics is the study of a substance’s absorption, distribution, metabolism, excretion, and toxicity (ADMET) inside the body [19]. ADMET features of particular chemicals in plant extracts can be anticipated using in silico methods that examine parameters such as oral bioavailability, blood–brain barrier penetration, and metabolic stability [20]. Key factors, such as molecular weight, lipophilicity (logP), hydrogen bond donors/acceptors, and polar surface area, are utilized to assess if these compounds follow Lipinski’s Rule of Five, which predicts drug-likeness [21]. Integrating molecular docking with pharmacokinetic predictions provides full knowledge of the potential of plant-derived drugs, directing future in vitro and in vivo research [22].

Notably, the biological properties of the root extracts have only been elucidated in a few preliminary studies in the field of *A. absinthium* research. The flowers, leaves, stems, and roots of *A. absinthium* extracts demonstrated antifungal [23], and potentially cytotoxic effects on the breast cancer cell line (MCF-7) [24]. However, there is still a noticeable lack of research on the antioxidant, antidiabetic, and antibacterial properties of root extract. Although a lot of material has been written on an extract of *A. absinthium*, our study closes a big gap since this plant has not been well studied scientifically, despite its well-known traditional therapeutic use. To the best of our knowledge, this is the first study that describes the chemical profiling, antioxidant capacity in vitro and in silico, anticancer, antibacterial, and antidiabetic properties of AARE. Furthermore, this work further elucidates the underlying processes that determine the anticancer of AARE by employing quantitative reverse transcriptase PCR (RT-qPCR)-based mRNA expression profiling of particular pro- and anti-apoptosis marker genes. Thus, the purpose of this research is to identify *A. absinthium’s* previously undiscovered medicinal properties using the root extract. An innovative method that reveals a distinct and rich chemical composition is the thorough chemical analysis using gas chromatography-mass spectrometry (GC-MS). In addition to measuring the amounts of total phenolic content (TPC) and total flavonoid content (TFC), this research provides strong proof of the extract’s antibacterial, anticancer, antidiabetic, and antioxidant properties. A thorough understanding of the extract’s pharmacological properties is also provided by the combination of chemical profiling, in vitro biological evaluations, and in silico research. These results provide fresh information on the possible pharmacological uses of *A. absinthium*, particularly its impact on causing apoptosis in cancer cell lines and its powerful antibacterial activity against different strains. Thus, this study’s goals were to assess AARE’s chemical profile, as well as antioxidant, anticancer, antibacterial, and antidiabetic properties in vitro and in silico.

## 2. Results

### 2.1. Extraction Yields

The AARE yielded 37.87 ± 1.29% results based on the dry matter weight calculation (*w*/*w*) and the applied operating mode.

### 2.2. Chemical Composition of AARE

**Figure 1:**
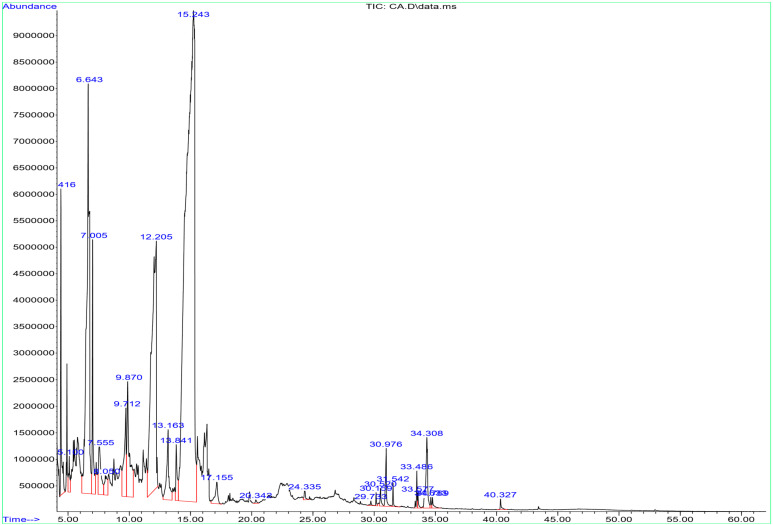
The GC-MS chromatograms of AARE. The GC-MS instrument was configured to run for 73 min with AARE for this experiment. All spectral peaks correlate with identified chemicals, with a big peak indicating the primary constituent of the extract.

The bioactive components of the AARE were investigated using GC-MS. Twenty-six peaks were identified by comparing the bioactive components of AARE with known compounds listed in the NIST library. These peaks were identified by peak retention time (RT), peak area (%), molecular formula (MF), and molecular weight (MW). As 51.79% of the total extract, the aldehyde of 5-hydroxymethylfurfural has been identified to be the main constituent, followed by 4H-Pyran-4-one, 1,5-Hexadien-3-ol (13.26%), and 2,3-dihydro-3,5-dihydroxy-6-methyl (14.35%) (Table 1 and Figure 1).

### 2.3. TPC and TFC of the AARE

The TPCs and TFCs of AARE were determined using colorimetric methods. The AARE was found to contain higher levels of TPC (97.34 ± 2.11 mg GAE/g of extract; R^2^ = 0.912) compared to TFC (47 ± 1.42 mg QE/g of extract with R^2^ = 0.941).

### 2.4. 1,1-.Diphenyl-2-picryl hydrazyl (DPPH) and 2,2′-azino-bis (3-ethylbenzothiazoline-6-Sulfonic Acid) (ABTS) Assay

**Figure 2:**
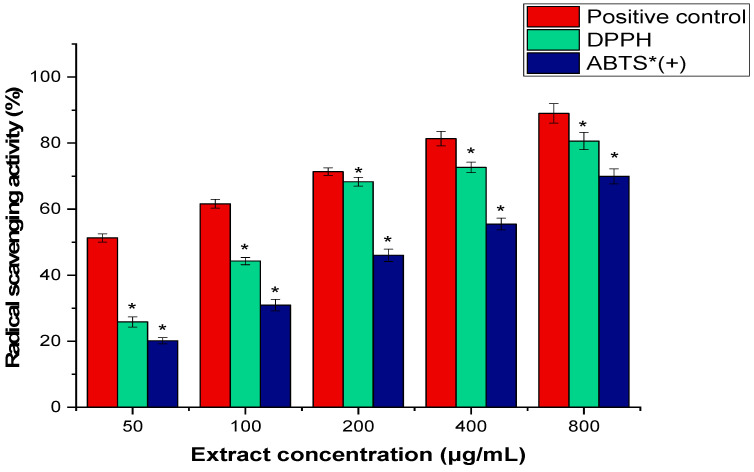
Antioxidant activity of AARE scavenging activity) at various concentrations (50–800 μg/mL). A positive control was an ascorbic acid. Three separate experiments were used to obtain the mean ± SD data. At a significance level of *p* < 0.05, the scavenging activity of AARE was substantially lower (*) than the positive control. + stands for radical cation.

The potent antioxidant activity of AARE was compared to ascorbic acid as a positive control using the DPPH and ABTS radical scavenging methods (Figure 2). In comparison to the positive control (IC_50_ value: 153.11 ± 1.14 μg/mL), the AARE exhibited a moderate scavenging activity for DPPH (IC_50_ value: 172.41 ± 3.15 μg/mL) and ABTS radicals (378.94 ± 2.18 μg/mL).

### 2.5. Cell Cytotoxicity

**Figure 3:**
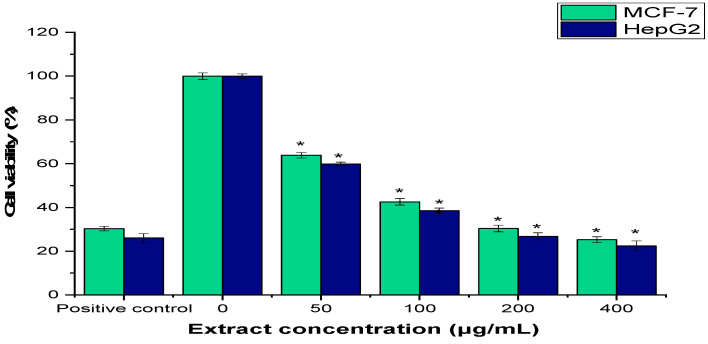
The impact of AARE on MCF-7 and HepG2 cell viability was assessed using the MTT assay. The cells were exposed to the AARE (0–400 μg/mL) for an entire day. The mean ± SD for the three different investigations is shown (* = *p* < 0.05 relative to the untreated cells (negative control)).

The anticancer efficacy of AARE against HepG2 (human liver cancer) and MCF-7 (human breast cancer) was evaluated using the 3-4,5-dimethylthazolk-2-yl)-2,5-diphenyl tetrazolium bromide (MTT) assay. The results indicate that the AARE decreased the viability of MCF-7 and HepG2 cancer cells in a dose-dependent manner. It is interesting to note that extract concentrations affect cell viability. Interestingly, cell viability decreased to 63.83 ±1.25% for MCF-7 cells and 59.81 ±0.85% for HepG2 cells, with the addition of 50 μg/mL of AARE. However, for the positive control (cisplatin 30 µg/mL), MCF-7 and HepG2 viability were 30.28 ± 1.09% and 26.05 ± 1.95%, respectively. The maximum cytotoxic activity was observed for MCF-7 and HepG2 cells at 400 μg/mL, with 25.24 ± 1.33% and 22.42 ± 2.25%, respectively. Overall, the AARE has shown moderated anticancer activity against MCF-7 cells, with IC_50_ values of 150.12 ± 0.74 μg/mL, and HepG2 cells, with IC_50_ values of 137.11 ± 1.33 μg/mL (Figure 3).

### 2.6. Apoptotic Analysis

**Figure 4:**
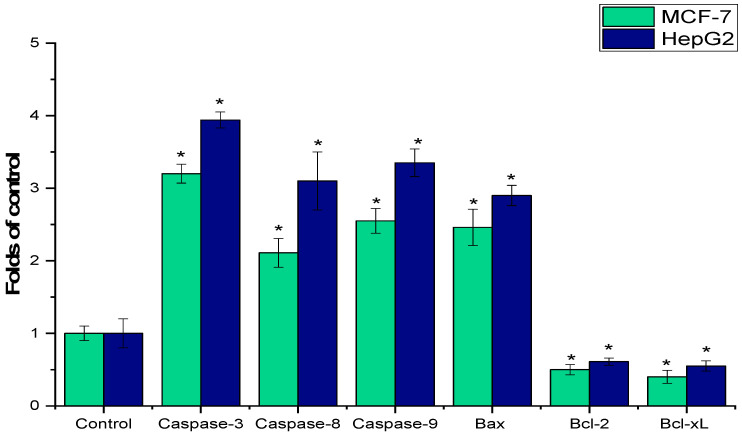
Effect of AARE (150 μg/mL) on MCF-7 and HepG2 cell lines and analysis of pro-apoptosis (*caspase-3, 8* and 9, and *Bax),* and anti-apoptosis marker *(Bcl-2* and *Bcl-Xl*) genes for 48 h. Gene expression (* = *p* < 0.05 relative to untreated cells, or control) is shown by the mean ± SD of three independent experiments.

The AARE was evaluated using RT-qPCR assay to assess its effects on induced apoptotic signaling in MCF-7 and HepG2 cells. Treatment with AARE resulted in a significant downregulation of anti-apoptotic genes, and an increase in the activity of apoptotic genes (*caspase-3*, *8*, *9*, and *Bax*) compared to the untreated control cells (*p* < 0.05) (Figure 4).

### 2.7. In Vitro α-Amylase and Glucosidase Inhibition Activities

**Figure 5:**
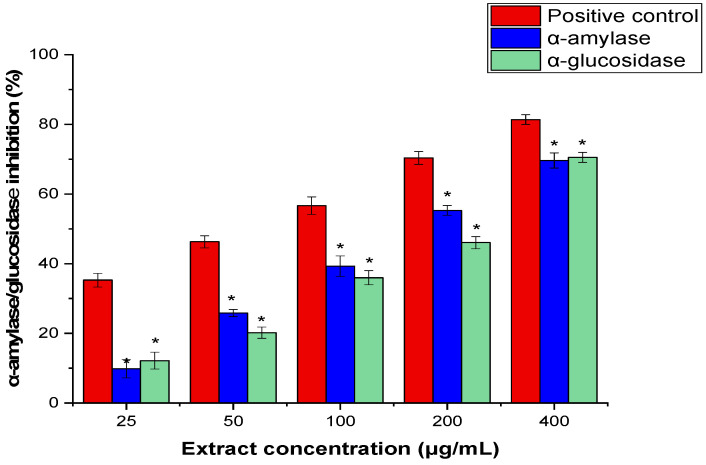
Effect of the ARE on *α*-Amylase and glucosidase inhibitory activities at various concentrations (25–400 μg/mL). The three independent experiments’ mean ± SD are the results (* = *p* < 0.05 concerning the positive control).

The AARE was evaluated for its potential to prevent diabetes using the α-amylase and α-glucosidase inhibitory tests. The results indicated a significant inhibitory action against both enzymes with IC_50_ values of 224.12 ± 1.17 μg/mL and 243.35 ± 1.51 μg/mL, respectively, as compared to the positive control, acarbose (*p* < 0.05) (Figure 5).

### 2.8. Antibacterial Effects of the AARE

The AARE was found to have dose- dependent antimicrobial effects on all tested bacteria. The data displayed in Table 2 show the antibacterial activity of AARE using disk diffusion, minimum inhibitory concentration (MIC), and minimum bactericidal concentration (MBC) techniques. Notably, Gram-positive bacteria (MIC values = 3.12 ± 0.00–6.25 ± 0.00 μg/mL) are more sensitive to AARE than Gram-negative bacteria (MIC values = 12.50 ± 0.00–25 ± 0.00 μg/mL) (Table 2).

### 2.9. Molecular Docking Analysis

The inhibition of NADPH oxidase is essential for maintaining cellular redox balance and enhancing antioxidant defense systems. Nicotinamide adenine dinucleotide phosphate (NADPH) is a critical cofactor in various enzymatic processes that support cellular antioxidant mechanisms. Antioxidants protect cells and tissues from oxidative damage caused by free radicals, highly reactive molecules capable of damaging DNA, proteins, and lipids, contributing to aging and various diseases. In our in silico analysis, three compounds were identified as potent inhibitors of NADPH oxidase: 2,4-Dihydroxy-2,5-dimethyl-3(2H)-furan-3-one, 2,5-Dimethylfuran-3,4(2H,5H)-dione, and Furaneol, with glide scores of −7.118, −6.461, and −6.440 kcal/mol, respectively (Table 3).

Regarding the antibacterial activity compound identified in AARE, 1H-Purin-2-amine, 6-methoxy-N-methyl- exhibited the highest inhibitory activity against *E. coli* Gyrase B, with a glide score of −6.929 kcal/mol. This was followed by Azuleno [4,5-b] furan-2(3H)-one, decahydro-3,6,9-tris(methylene)-, and 2,4-Dihydroxy-2,5-dimethyl-3(2H)-furan-3-one, with glide scores of −6.233 and −6.057 kcal/mol, respectively (Table 3).

Concerning anticancer activity of AARE, 4H-Pyran-4-one, 2,3-dihydro-3,5-dihydroxy-6-methyl-, 4-Pyridinecarboxylic acid, 3-hydroxy-5-(hydroxymethyl)-2-methyl, and 2,4-Dihydroxy-2,5-dimethyl-3(2H)-furan-3-one showed strong affinity to Topoisomerase IIα, with glide scores of −11.030, −10.798, and −10.796 kcal/mol, respectively (Table 3).

**Figure 6:**
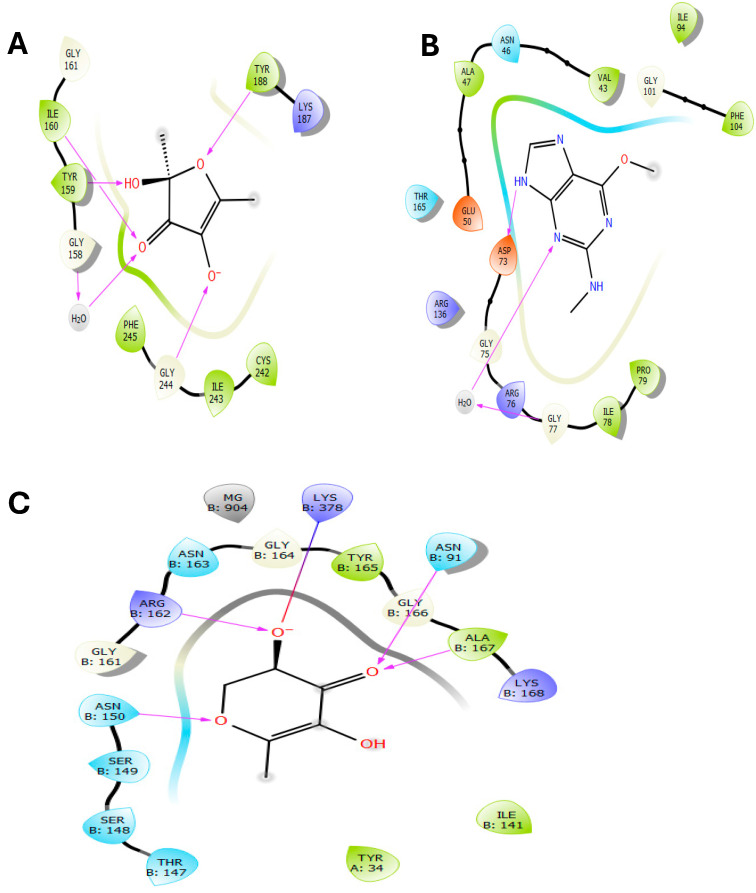
The 2D viewer of ligands interactions with the active site. (**A**): 2,4-Dihydroxy-2,5-dimethyl-3(2H)-furan-3-one interactions with the active site of NADPH oxidase. (**B**): 1H-Purin-2-amine, 6-methoxy-N-methyl- interactions with the *E. coli* gyrase B site active. (**C**): 4H-Pyran-4-one, 2,3-dihydro-3,5-dihydroxy-6-methyl- interactions with the active site of Topoisomerase IIα.

**Figure 7:**
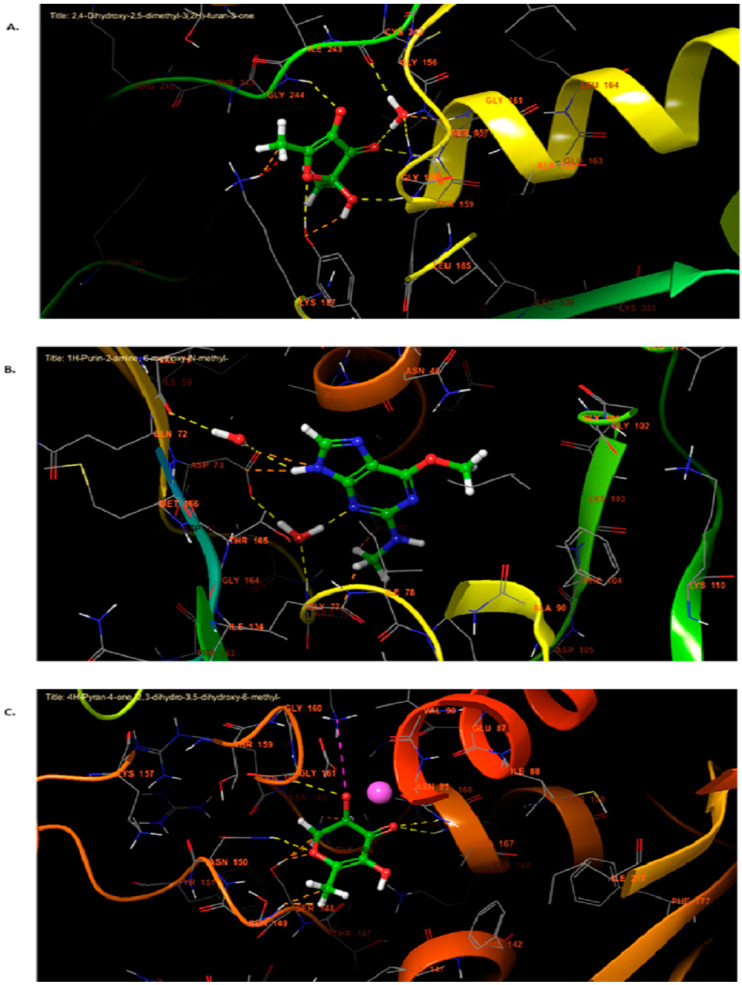
The 3D viewer of ligands interactions with the active site. (**A**): 2,4-Dihydroxy-2,5-dimethyl-3(2H)-furan-3-one interactions with the active site of NADPH oxidase. (**B**): 1H-Purin-2-amine, 6-methoxy-N-methyl- interactions with the *E. coli* gyrase B site active. (**C**): 4H-Pyran-4-one, 2,3-dihydro-3,5-dihydroxy-6-methyl- interactions with the active site of Topoisomerase IIα.

The 2D and 3D interaction analysis of the NADPH oxidase active site with ligands revealed that 2,4-Dihydroxy-2,5-dimethyl-3(2H)-furan-3-one formed five hydrogen bonds with residues ILE 161, ILE 160, TYR 159, GLY 244, and TYR 188 (Figure 6A and Figure 7A). These strong interactions contribute to its high binding affinity, making it our study’s most potent NADPH oxidase inhibitor.

In the active site of *E. coli,* Gyrase B, 1H-Purin-2-amine, and 6-methoxy-N-methyl- interact via two hydrogen bonds with the ASP 73 and GLY 77 residue (Figure 6B and Figure 7B), highlighting its specific interaction with critical amino acids for enzyme function.

Interaction analysis revealed that 4H-Pyran-4-one, 2,3-dihydro-3,5-dihydroxy-6-methyl- formed four hydrogen bonds with residues ASN B: 150, ARG B: 162, ASN B: 91, and ALA B: 167, and established a salt bridge with LYS B: 378 in the active site of Topoisomerase IIα (Figure 6C and Figure 7C). These interactions suggest strong binding within the Topoisomerase IIα active site, which could significantly disrupt its catalytic activity and lead to potential anticancer effects.

### 2.10. Prediction of ADMET by Computational Analysis

The molecule 2,4-Dihydroxy-2,5-dimethyl-3(2H)-furan-3-one emerged as a potent inhibitor of NADPH oxidase in the in silico study. From a pharmacokinetic perspective, 2,4-Dihydroxy-2,5-dimethyl-3(2H)-furan-3-one has an MW of 144.127 Da, making it small and likely to permeate cellular membranes efficiently. Its SASA of 259.514 Å^2^ indicates a reasonable surface area for interaction with biological targets. The predicted Caco-2 cell permeability (2074.855 nm/s) and %HOA (84.363%) suggest that this compound is well absorbed orally, making it a strong candidate for further development as an antioxidant agent. Additionally, its blood–brain barrier penetration potential (QPlogBB = −0.032) suggests it could have peripheral effects without crossing into the central nervous system. It is desirable for compounds targeting oxidative stress without neurological implications. For antibacterial activity, 1H-Purin-2-amine, 6-methoxy-N-methyl- was identified as a potent inhibitor of *E. coli* Gyrase B. Pharmacokinetically, this compound has an MW of 183.163 Da and an SASA of 320.421 Å^2^, indicating a balance between size and interaction surface area. It features two hydrogen bond donors and four hydrogen bond acceptors, which is within the acceptable range. Its predicted Caco-2 permeability (1593.16 nm/s) is substantial, indicating high absorption through the intestinal wall, while its %HOA (70.891%) also confirms good oral bioavailability. The QPlogBB value of 0.218 suggests that 1H-Purin-2-amine could cross the blood–brain barrier, making it a candidate for further research in peripheral and central antibacterial applications. However, additional safety evaluations regarding neurotoxicity would be necessary. The molecule, 4H-Pyran-4-one, 2,3-dihydro-3,5-dihydroxy-6-methyl-, demonstrated significant anticancer potential by inhibiting Topoisomerase IIα. This molecule has a molecular weight of 144.127 Da, making it ideal for cellular penetration. Its SASA of 342.706 Å^2^ provides adequate surface area. With one hydrogen bond donor and four acceptors, it aligns with the drug-likeness guidelines, promoting favorable interactions with biological targets. The predicted Caco-2 permeability (905.191 nm/s) suggests moderate but sufficient intestinal absorption, while its %HOA (78.38%) indicates good oral bioavailability. Additionally, its QPlogBB value of -0.658 suggests limited blood–brain barrier permeability, which may be beneficial for targeting peripheral cancer cells without risking central nervous system side effects (Table 4). The pharmacokinetic evaluation of the critical molecules identified in the docking studies (2,4-Dihydroxy-2,5-dimethyl-3(2H)-furan-3-one, 1H-Purin-2-amine, 6-methoxy-N-methyl-, and 4H-Pyran-4-one, 2,3-dihydro-3,5-dihydroxy-6-methyl-) demonstrates that they possess favorable properties for drug development. All three compounds exhibit strong binding affinity to their respective targets—NADPH oxidase, *E. coli* Gyrase B, and Topoisomerase IIα—along with good predicted oral bioavailability, permeability, and compliance with Lipinski’s drug-likeness criteria. The limited blood–brain barrier penetration observed for most of these compounds suggests they are suitable for non-neuroactive therapeutic applications. Given these promising results, further in vitro and in vivo validation studies are warranted to explore their full therapeutic potential.

## 3. Discussion

Traditionally, root extracts have been valued for their distinct bioactive compounds, many of which may be absent or in lower concentrations in leaves [25]. Many bioactive chemicals in roots are kept in stable forms, which can help them last longer and be more effective when extracted. Roots are frequently less exposed to environmental stresses that might destroy certain phytochemicals. Root extracts have been utilized for millennia in various cultures (such as the Chinese ginger as a tonic root), and their efficiency and safety profiles have been proven through historical use [26]. This traditional understanding contributes to their ongoing usage in modern herbal treatment. In their pre-clinical trial, Kauser et al. (2023) revealed that the ethanolic extract of *Artemisia absinthium* showed the least short-lived signs of toxicity in rats at 300 mg/kg, where the dose of 50 mg/kg is considered mostly safe [27]. These findings emphasize the potential of root extracts in both traditional and modern medicinal uses, providing a viable path for further study into their bioactive components and mechanisms of action in clinical settings.

In the current study, we used an in vitro and in silico approach to discover and analyze the bioactive components in the root extract. Our in vitro assays, which include cytotoxicity, antioxidant, and anti-inflammatory testing, aid in determining the safety and effectiveness of the discovered compounds. Furthermore, we used molecular docking and other in silico approaches to identify possible interactions with major biological targets, allowing us to better understand the molecular basis of their impacts. This multimodal method, which combines chemical profiling with biological and computational analysis, allows us to find promising chemicals and distinguish between helpful and possibly dangerous substances.

In the current study, the methanolic extraction of AARE parts was used. The AARE yielded 37.87 ± 1.29%. The predicted yield of methanolic extraction of AARE is determined by various factors, including the plant portion employed, the drying technique, the solvent concentration, extraction time, and other processing parameters. Yield is often stated as a percentage of the plant’s dry weight. That yields a wide range of bioactive chemicals, including phenolic acids, flavonoids, and sesquiterpene lactones [28]. Several studies compared the use of different solvents in the extraction of different plant parts of *A. absinthium*. In that context, a previous study showed that the methanolic extraction of *A. absinthium* aerial parts yielded 11.6% using absolute methanol [29]. In the study conducted by Sofi et al. (2022), the methanolic and ethanolic extraction of *A. absinthium* leaves resulted in 9.793% and 7.753%, respectively [30]. Another study showed that the aqueous and ethyl acetate extracts of *A. absinthium* leaves yielded 0.68% and 16%, respectively [12]. Trifan et al. used both methanol and chloroform in the extraction procedure of *A. absinthium* roots and ariel parts and showed that methanol was more efficient in producing the highest yield using 10 g of the dried material [4]. This implies that methanol is still the best solvent for extracting bioactive chemicals from *A. absinthium*, with a higher yield than other solvents across different plant sections.

Plant components with an aromatic ring that contains one or more hydroxyl groups are known as phenolic compounds. The number of naturally occurring plant phenolics is around 8000. Plant components include flavonoids, a major class of naturally occurring phenolic chemicals, both in their free form and as glycosides. Of all the several subclasses of phenolics, flavonoids are the most prevalent and varied class of phenolic chemicals found in nature. These substances occur in both free and glycoside forms in a variety of plant tissues. Flavonoids’ importance in enhancing the TPC and antioxidant capability of plants is further shown by their occurrence in a variety of plant parts [31]. The solubility of phenolic compounds is influenced by solvent polarity, which may impact extraction yield and activity due to their variable chemical composition [32]. While phenolic acids and catechin are more effectively extracted with methanol, flavonoids, and their glycosides are more efficiently extracted using methanol [33]. Another investigation tested the TPC and TFC of the extracts of *A. absinthium* leaves using ethyl acetate, methanol, and water as the three solvents. Of the three extracts, the most TPC was found in the water extract (134.47 mg 100g DW^−1^), which was followed by methanol (131.18 mg QE/g of extract) and ethyl acetate (51.49 mg QE/g of extract). The range of TFC was 18.85–87.04 mg QE/g of extract. In the present study, the colorimetric analysis of the methanolic extract of AARE demonstrated a high concentration of phenolic components (TPC = 97.34 ± 2.11 mg GAE/g of extract). This was significantly greater than the TFC, which measured 47 ± 1.42 mg QE/g of extract. The findings are consistent with prior studies, showing that the TPC of the aerial part of *A. absinthium* L. collected from Iran during the flowering stage was greater (194.9 9.7 mg GAE/g of extract) than the TFC (12.4 0.6 mg QE/g of extract) [34]. A recent study reported that the TPC of methanolic extracts and essential oils of aerial parts of *A. absinthium* from five different regions in Tunisia (Bizerte, Zaghouan, Kasserine, Gabes, and Tozeur) varied significantly (*p* ≤ 0.05) from region to region (6.93 ± 0.00 mg GAE/g of extract to 26.80 ± 0.00 mg GAE/g of extract); however, the highest TFC was detected in Zaghouan region (6.84 ± 0.02 mg QE/g of extract) [35]. The high TPC suggests that the methanolic extract includes a significant quantity of phenolic acids, which are known to have strong antioxidant capabilities. Flavonoids, including quercetin and kaempferol, are renowned for their anti-inflammatory, antibacterial, and anticancer activities [36]. Although flavonoids are less prevalent than phenolics in AARE, they help to enhance the extract’s bioactivity. This balance of phenolic and flavonoid content may explain the plant’s many biological effects, including its usage as an anti-inflammatory and antibacterial agent [37]. The increased TPC relative to TFC indicates that phenolic acids, such as caffeic acid, chlorogenic acid, and 5-hydroxymethylfurfural, are more important in the root’s bioactivity than flavonoids. Similar investigations have shown that *A. absinthium* has a greater phenolic content than flavonoid content in various solvent extractions [4,28]. Furthermore, the correlation values (R^2^) of 0.912 for TPC and 0.941 for TFC indicate a significant linear association between phenolic and flavonoid concentrations in the extract and their colorimetric responses. This excellent correlation confirms the dependability of the colorimetric techniques utilized and suggests that the obtained values accurately reflect the extract’s phenolic and flavonoid content [38]. In comparison to other plant components, AARE appears to exhibit specific chemical properties. The leaves of *A. absinthium* have a total flavonoid concentration of 3.80 ± 0.13%, with the major components being Astragalin, Cynaroside, Ononin, Rutin, Kaempferol-3-O-rutinoside, Diosmetin, Isorhamnetin, and Luteolin [37]. Trifan et al. showed that aerial parts of *A. absinthium* have greater flavonoid concentration than roots, suggesting that the plant component employed in extraction has a major impact on the bioactive profile [4]. Furthermore, the use of methanol as an extraction solvent has been shown to favor the extraction of phenolics over flavonoids, which may explain the observed greater TPC in the current investigation [28]. Overall, the high TPC of AARE implies that it has the potential to be a powerful antioxidant source, which might aid in its therapeutic usage in the treatment of oxidative stress and associated illnesses. Meanwhile, the high flavonoid content enhances its anti-inflammatory and antibacterial effects, emphasizing the root extract’s multifaceted advantages.

The GC-MS study of the methanolic extract of AARE revealed twenty-six bioactive components, with 5-hydroxymethylfurfural being the most abundant, accounting for 51.79% of the total extract. 5-hydroxymethylfurfural is a significant bioactive component with antioxidant, anti-inflammatory, and antibacterial activities that may add to AARE’s overall therapeutic potential [39]. Previous research has shown similar results, identifying 5-hydroxymethylfurfural as a substantial component in several plant extracts and associating it with possible health advantages such as anticancer and cardioprotective properties [40,41]. In their study conducted in India, Sofi et al. (2022) showed similar findings of the GC-MS analysis of ethanolic root extract of *A. absinthium,* where their analysis did not recognize any thujones and thujoneols volatile compounds [30], which are commonly detected in the leaves extract. That might indicate the unique chemical composition of the root extracts of *A. absinthium* species in Saudi Arabia.

The finding of 5-hydroxymethylfurfural as the major component raises fascinating questions about the extract’s bioactivity. 5-hydroxymethylfurfural, which is recognized for its bioactivity, may also occur as a result of the Maillard reaction during drying and extraction procedures, especially at high temperatures [42]. This shows that the extraction conditions, notably the temperature, may have altered the amount of 5-hydroxymethylfurfural in the methanolic extract, potentially affecting its biological activity. The second major component found was 4H-Pyran-4-one, which accounted for 13.26% of the extract. This chemical has been shown to have antibacterial, antioxidant, and anticancer properties, lending credence to *A. absinthium*’s traditional usage in the treatment of infections and inflammation [43,44]. The presence of 1,5-Hexadien-3-ol and 2,3-dihydro-3,5-dihydroxy-6-methylpyran in smaller amounts (14.35%) highlights AARE’s potential as an antioxidant source, supporting previous research that identified flavonoids and phenolic compounds in wormwood as key contributors to its medicinal properties [2,3]. Other investigations have shown a variety of bioactive components in *A. absinthium*, depending on the extraction process, solvent type, and plant section employed. For example, Ghafoori et al. and Dane et al. observed diverse sets of bioactive components, with terpenoids and sesquiterpene lactones dominating extracts of *A. absinthium* aerial parts [28,29]. The variance in compound profiles revealed in the current study suggests that aerial root extract may have a different chemical makeup than more regularly investigated components like leaves and flowers. Furthermore, the antimicrobial and anti-inflammatory properties of some of the discovered chemicals are consistent with *A. absinthium*’s traditional folk medicine usage, notably for the treatment of digestive and inflammatory problems. Hbika et al. and Trifan et al. verified that several solvent extracts of *A. absinthium* exhibited high antibacterial activity [4,12], which might be attributable to the presence of chemicals such as 5-hydroxymethylfurfural and pyranones, as discovered in our investigation. These findings also lend evidence to wormwood’s potential as a natural antibacterial and antioxidant agent. The root extract’s specific chemical profile, as opposed to aerial parts, implies that it might be used in innovative medicinal or nutraceutical compositions.

The most widely used methods for determining antioxidant capacity are DPPH and ABTS because of their simplicity, speed, sensitivity, and use of stable radicals [45]. The DPPH is based on the reduction in a methanolic DPPH solution in the presence of an antioxidant that donates hydrogen as a result of the reaction, producing the non-radical form DPPH-H. The extract was able to reduce the stable radical DPPH to the yellow-colored diphenylpicrylhydrazine [46]. The discrepancy in activity between the DPPH and ABTS tests can be attributed to the different processes involved in radical scavenging [47]. The ABTS radical cation method is a nitrogen-centered synthetic radical cation produced by oxidizing ABTS with potassium per-sulfate. To transform the ABTS radical dot into its non-radical form, an antioxidant component may contribute an electron [48]. AARE’s IC_50_ values for DPPH and ABTS radical scavenging were 172.41 ± 3.15 μg/mL and 378.94 ± 2.18 μg/mL, respectively, compared to 153.11 ± 1.14 μg/mL for ascorbic acid. The higher IC_50_ value for ABTS in the AARE may indicate that AARE includes chemicals that are more effective in hydrogen donation than electron donation. A recent study found that the essential oil of *A. scoparia* has substantial antioxidant effects in free radical scavenging tests for DPPH (IC_50_ = 285 ± 0.82 µg/mL) and ABTS (IC_50_ = 295 ± 0.32 µg/mL) [49]. The modest antioxidant activity of AARE can also be linked to the bioactive components discovered during GC-MS research, such as 5-hydroxymethylfurfural and 4H-Pyran-4-one, which have been shown to have antioxidant characteristics, contribute to the free radical scavenging activity demonstrated in this study [41,44]. The inclusion of phenolic acids in the TPC assay adds to the moderate antioxidant activity, as these chemicals are known to be effective radical scavengers. In compliance with these findings, a previous study evidenced the antioxidant activity of 24.00 mg Trolox equivalent per gram of *A. absinthium* essential oil [50]. Controversially, in the study conducted by Ali et al., the ferric reducing antioxidant power (FRAP) activity of combined plant extracts of *A. absinthium* leaves and *Citrus paradisi* peels was higher than their single effect, unlike the DPPH-radical scavenging activity [51]. Furthermore, Ghafoori et al. discovered that various sections of *A. absinthium* had varied levels of antioxidant activity based on their phenolic and flavonoid content [28], which supports the current study’s findings.

The MTT test was used to evaluate the anticancer activity of AARE against MCF-7 and HepG2 cells, and both cell lines showed a dose-dependent reduction in viability. MCF-7 and HepG2 showed IC_50_ values of 150.12 ± 0.74 μg/mL and 137.11 ± 1.33 μg/mL, respectively. These data indicate that AARE has moderated anticancer efficacy, especially when compared to the positive control, cisplatin (30 μg/mL). Cisplatin is currently the treatment of choice for many types of cancer, such as testis, over, bladder, prostate, cervical, and lung cancer. Cisplatin exerts anticancer activity via multiple mechanisms. Its most well-known methods, which eventually lead to death, include activating several signal transduction pathways, shutting or activating various genes, and creating DNA–platinum adducts via interactions with purine bases. Nevertheless, side effects and drug resistance are two inherent issues that limit the usage and effectiveness of cisplatin. Inactivating drugs via interactions with glutathione and metallothioneins diminishes drug accumulation in cancer cells and speeds up DNA lesion repair, which leads to cisplatin resistance [52]. AARE had somewhat stronger activity against HepG2 cells compared to MCF-7 cells, as evidenced by a lower IC_50_ value. This might be linked to the diverse phytochemical makeup, notably the existence of bioactive chemicals. These chemicals are recognized to have anticancer characteristics because they decrease cancer cell growth, induce apoptosis, and prevent angiogenesis [53]. Furthermore, AARE’s moderate antioxidant activity may contribute to its anticancer properties, as oxidative stress regulation is often connected to cancer cell survival and death [51]. An early study revealed that *A. absinthium* methanolic extract at 25 μg/mL over 50% reduced the development of breast cancer cells (MCF-7 and MDA-MB-231) in comparison to controls [54]. Previous research also has demonstrated that *Artemisia* species extracts exhibit comparable dose-dependent cytotoxicity against a variety of cancer cell lines. *A. absinthium* total flavonoids and the extracted fractions of Cynaroside and Astragalin exhibited cytotoxic effects against Hela cells at IC_50_ of 396.0 ± 54.2 μg/mL and 449.0 ± 54.8 μg/mL, respectively [37]. Also, the methanolic leaf extract of *A. absinthium* had antiproliferative activity against hepatocellular carcinoma (HUH-7 cells) [51,55], lung carcinoma (A549 and Calu-6) [11,56], colon adenocarcinoma (DLD1 and HCT116), and endometrium (ECC-1) cancer cells [7,57] with varied IC_50_ values based on extraction process and solvent. The obtained findings show the potential use of AARE as a natural anticancer drug.

Apoptosis is the primary mechanism that is essential for maintaining a balance between cell division and growth to prevent uncontrolled malignancy [58]. The majority of anticancer medications cause cancer cell death by taking advantage of intact apoptotic signaling pathways [59]. The capacity of AARE to induce apoptosis in MCF-7 and HepG2 cells was studied using RT-qPCR, which revealed considerable modification of both pro-apoptotic and anti-apoptotic genes. AARE treatment increased apoptotic genes (*caspase-3*, *caspase-8*, *caspase-9*, and *Bax*) and decreased anti-apoptotic genes compared to untreated control cells. The increase in caspases in response to AARE indicates that the extract triggers caspase-mediated apoptosis, which results in cancer cell death. A previous study found that 25 μg/mL aerial parts of *A. absinthium* extract activated caspase-7 and elevated Bcl-2 protein in breast cancer cells (MCF-7 and MDA-MB-231) [54]. Extrinsic (mediated by death receptors), intrinsic (mediated by mitochondria), and endoplasmic reticulum stress-dependent (ERS) signaling transduction are the three primary mechanisms that lead to apoptosis. Through the activation of the executioner caspases -3, -6, and -7, caspase-8 is an essential facilitator of the extrinsic pathway that initiates apoptosis. Caspases -3 and -7 are essential upstream mediators in the intrinsic route that initiates apoptosis. It was discovered that caspase-3 exhibited both endogenous and external apoptotic characteristics via interactions with caspase-8 and -9 [60]. Furthermore, increased expression of Bax, a pro-apoptotic member of the Bcl-2 family, suggests that AARE enhances mitochondrial membrane permeabilization, which drives cells to death [61]. Anti-apoptotic genes, such as *Bcl2*, block apoptosis by preserving mitochondrial membrane integrity. Therefore, the Bax/Bcl2 ratio determines the sensitivity to apoptotic stimuli [62]. The downregulation of anti-apoptotic genes in both MCF-7 and HepG2 cells lends credence to the theory that AARE disrupts cancer cell survival pathways, leaving them more vulnerable to apoptotic triggers. The regulation of these pathways is an important therapeutic technique in cancer treatment since many cancer cells are resistant to apoptosis due to overexpression of anti-apoptotic proteins such as Bcl-2 [63]. In compliance with our findings, a previous study revealed that the methanolic extract of *A. absinthium* increased the expression levels of BAX and caspases, where it decreased those of Bcl-2 genes in human colorectal cancer HCT-116 cells [64]. Also, Dane et al. (2016) showed that *A. absinthium* ethanol extract upregulated the expression levels of cytochrome c, caspase-3, caspase-9, and PARP in the human hepatoma BEL-7404 cells and mouse hepatoma H22 cells. These findings emphasize AARE’s potential therapeutic implications in cancer treatment, notably as a natural anticancer drug that promotes caspase-mediated apoptosis [29].

Since the medications now used to treat hyperglycemia are toxic and have unfavorable side effects, researchers are searching for novel intestinal α-glucosidase and pancreatic α-amylase inhibitors from natural sources, particularly plants, that have a hypoglycemic impact with few or no adverse effects. Numerous plants that function as enzyme inhibitors have been investigated for their potential to cure diabetes [65]. AARE’s antidiabetic efficacy was tested by analyzing its inhibitory effects on α-amylase and α-glucosidase enzymes, which play critical roles in carbohydrate digestion and modulate type 2 diabetes mellitus (T_2_DM) [66]. The study found that AARE significantly inhibited both enzymes, with IC_50_ values of 224.12 ± 1.17 μg/mL for α-amylase and 243.35 ± 1.51 μg/mL for α-glucosidase, when compared to the positive control, acarbose, a clinically utilized antidiabetic drug. AARE’s substantial inhibitory impact on both enzymes implies that it might be used as a natural antidiabetic drug, matching the actions of synthetic inhibitors such as acarbose. In agreement with our findings, Ghafoori et al. found that *A. absinthium* extracts had strong antidiabetic action, with inhibitory effects equivalent to conventional medicines in both in vitro and in vivo models [28]. Another study showed that the extracts of the Ariel part of *A. absinthium* inhibited both α-Amylase and α-glucosidase IC_50_ to 19.4 ± 0.5 and 31.90 ± 1.8 μg/mL, respectively [13]. Interestingly, Hbika et al. reported that the aqueous extracts of *A. absinthium* were able significantly to inhibit α-Amylase and α-glucosidase, in vitro and STZ-diabetic rat models [12]. α-Amylase and α-glucosidase can be inhibited by *A. absinthium’s* bioactive compounds, such as flavonoids, terpenoids, and other secondary metabolites [67]. These phytochemicals attach to the enzyme’s active site or interact with its allosteric sites, preventing the enzyme from catalyzing the hydrolysis of starch into sugar [68,69]. Furthermore, Daradka et al. demonstrated that *A. absinthium* extracts exert their hypoglycemic effect by stimulating the AMP-activated protein kinase (AMPK) pathway, which is mainly due to the Thujyl alcohol, α- and β-thujones, azulenes, cadinene, bisabolene, sabinene, phellandrene, pinene components reported in their chemical composition [70]. The current findings and previous studies highlight the potential of AARE as a supplemental therapy for diabetic control.

The antibacterial ability of AARE was tested against a variety of bacterial strains utilizing disk diffusion, MIC, and MBC methods. The findings revealed a dose-dependent suppression of bacterial growth, with AARE exhibiting broad-spectrum antibacterial action against both Gram-positive and Gram-negative bacteria. Gram-positive bacteria were more vulnerable to AARE, with MIC values ranging from 3.12 ± 0.00 to 6.25 ± 0.00 μg/mL, whereas Gram-negative bacteria were more resistant, with MIC values ranging from 12.50 ± 0.00 to 25 ± 0.00 μg/mL. These findings indicate that AARE is more efficient in inhibiting Gram-positive bacteria than Gram-negative ones. The considerable antibacterial activity reported, notably against Gram-positive bacteria, may be due to the cell wall composition of these pathogens. Gram-positive bacteria have a simpler cell wall structure made up of a thick peptidoglycan layer that may be more easily pierced by the active chemicals in AARE. In contrast, Gram-negative bacteria have an outer membrane that functions as a barrier, making them more resistant to various antimicrobial treatments [71,72]. That increases the sensitivity of Gram-positive bacteria to plant-based extracts rich in phenolic acids, flavonoids, and sesquiterpene lactones. These chemicals may exert their antibacterial actions in a variety of ways, including damaging bacterial cell walls, blocking key enzymes, or interfering with DNA synthesis [36,73]. The presence of 5-hydroxymethylfurfural, a key component in AARE, may potentially contribute to its antimicrobial action [74]. Several studies reported different antibacterial activities of various *A. absinthium* extracts. Mohammed et al. showed that the ethyl acetate extract of *A. absinthium* had significant inhibitory effects against Hospital acquired *Acinetobacter baumannii, S. aureus,* and *Salmonella enteritidis,* while it did not affect the bacterial growth of the Gram-negative *Shigella boydii* [50]. Another study showed that the methanol extracts of *A. absinthium* had a broad-spectrum antibacterial activity against Gram-positive bacteria including *S. aureus*, *S. epidermidis*, *P. aeruginosa*, and *B. cereus* with *S. epidermidis* being the most sensitive among the tested bacteria [66]. A previous report showed that *A. absinthium* extracts are rich in different monoterpenes, including α-Thujone, β-Thujone, and Myrcene, which enable them to exert their potential antibacterial activities [75]. The current study supports previous findings by demonstrating the potential of AARE as a natural antibacterial agent, particularly against Gram-positive organisms.

The molecular docking studies for AARE’s bioactive chemicals provide interesting insights into their antioxidant, antibacterial, and anticancer properties. In silico docking of these drugs against major biological targets revealed strong inhibitors of NADPH oxidase, DNA Gyrase B, and Topoisomerase IIα, each with distinct therapeutic effects. Inhibiting NADPH oxidase is crucial for regulating oxidative stress, which has been associated with a variety of illnesses, such as aging, cardiovascular problems, and cancer [76]. The molecule 2,4-Dihydroxy-2,5-dimethyl-3(2H)-furan-3-one has the greatest binding affinity for NADPH oxidase, with a glide score of -7.118 kcal/mol, indicating a high potential as an antioxidant. Its capacity to make several hydrogen bonds with residues in the active region of NADPH oxidase (ILE 161, ILE 160, TYR 159, GLY 244, and TYR 188) supports the concept of efficient enzyme inhibition. The result is consistent with the fact that *A. absinthium* includes a high concentration of phenolic compounds and flavonoids, which are known to have strong antioxidant properties [28]. The high glide score indicates that 2,4-Dihydroxy-2,5-dimethyl-3(2H)-furan-3-one may help avoid oxidative damage, which supports its potential use in the development of therapies for oxidative stress-related disorders.

The antibacterial activity of AARE was assessed by docking its components against *E. coli* Gyrase B, a well-known target for bacterial inhibition [77]. The chemical 1H-Purin-2-amine, 6-methoxy-N-methyl- had the highest inhibitory activity, with a glide score of −6.929 kcal/mol. It formed two hydrogen bonds with critical residues ASP 73 and GLY 77 in the enzyme’s active site. These interactions are required to limit Gyrase B’s ATPase activity, hence preventing DNA supercoiling and bacterial replication. The efficiency of 1H-Purin-2-amine, 6-methoxy-N-methyl- in suppressing Gyrase B is consistent with the extensive antibacterial characteristics of *Artemisia* species described in earlier research [3]. The compound’s high binding affinity for bacterial targets makes it a promising option for future investigation as an antibacterial agent, especially against Gram-negative bacteria.

Topoisomerase IIα, an enzyme involved in DNA replication and repair, is frequently overexpressed in rapidly reproducing cancer cells, making it an appealing target for anticancer therapy [78]. AARE compounds (4H-Pyran-4-one, 2,3-dihydro-3,5-dihydroxy-6-methyl-, 4-Pyridinecarboxylic acid, 3-hydroxy-5-(hydroxymethyl)-2-methyl, and 2,4-Dihydroxy-2,5-dimethyl-3(2H)-furan-3-one) bind strongly to Topoisomerase IIα, with glide scores of −11.030, −10.798, and −10.796 kcal/mol, respectively. The interaction study found that 4H-Pyran-4-one, 2,3-dihydro-3,5-dihydroxy-6-methyl- established four hydrogen bonds with residues ASN B: 150, ARG B: 162, ASN B: 91, and ALA B: 167, as well as a salt bridge with LYS B: 378, indicating significant interactions inside the enzyme’s active site. These findings indicate that AARE chemicals can inhibit Topoisomerase IIα, causing DNA damage and potentially leading to death in cancer cells. The results are consistent with earlier studies that *A. absinthium* has anticancer properties [9,55]. AARE’s great binding affinities emphasize its promise in cancer therapy, especially for aggressive cancer forms that overexpress Topoisomerase IIα.

The pharmacokinetic assessment of the main compounds—2,4-Dihydroxy-2,5-dimethyl-3(2H)-furan-3-one, 1H-Purin-2-amine, 6-methoxy-N-methyl-, and 4H-Pyran-4-one, 2,3-dihydro-3,5-dihydroxy-6-methyl-—showed promising features for therapeutic development. All three drugs had strong oral bioavailability, as evidenced by high Caco-2 permeability and higher human oral absorption. Furthermore, their limited blood–brain barrier penetration indicates that they are appropriate for non-neuroactive therapeutic uses, lowering the danger of central nervous system adverse effects. The chemical 2,4-Dihydroxy-2,5-dimethyl-3(2H)-furan-3-one has a molecular weight of 144.127 Da and a surface area (SASA) of 259.514 Å^2^. Its small size and good permeability make it an attractive candidate for antioxidant research [79,80]. Similarly, 1H-Purin-2-amine, 6-methoxy-N-methyl- has significant antibacterial characteristics and a molecular weight of 183.163 Da, making it excellent for cellular penetration and contact with bacterial targets. Finally, 4H-Pyran-4-one, 2,3-dihydro-3,5-dihydroxy-6-methyl- shown promising anticancer characteristics, including substantial binding affinity to Topoisomerase IIα and favorable pharmacokinetics [81].

The present study offers novel insights into the therapeutic potential of AARE, focusing on its bioactive content and complex pharmacological activity. Unlike most earlier studies, which concentrated on the aerial parts and roots of *A. absinthium*, this is the first study that describes the chemical profiling of AARE. It reveals a unique chemical profile that is dominated by 1,5-Hexadien-3-ol, 4-H-Pyran-4-one, 5-hydroxymethylfurfural, and 2,3-dihydro-3,5-dihydroxy-6-methyl. The finding of its significant antioxidant by moderate DPPH and ABTS radical scavenging activity, strong anticancer action against MCF-7 and HepG2 cells, inducing apoptosis by upregulating pro-apoptotic genes while downregulating anti-apoptotic markers, antidiabetic by inhibiting α-amylase and α-glucosidase enzymes, which cause post-prandial hyperglycemia, and antibacterial activity, especially against Gram-positive bacteria, verified by both in vitro experiments and molecular docking investigations, adds to *A. absinthium’s* medical uses. The discovery of substances such as 2,4-Dihydroxy-2,5-dimethyl-3(2H)-furan-3-one as a potent NADPH oxidase inhibitor and 1H-Purin-2-amine as an efficient Gyrase B inhibitor is a big step forward in the hunt for natural antioxidants, and antibacterial. The study demonstrates that AARE species in Saudi Arabia has substantial anticancer effects on MCF-7 and HepG2 cells via inducing apoptosis and inhibiting Topoisomerase IIα. This is a fresh addition to the rising interest in plant-based cancer treatments.

We acknowledge that the approach we used in this research has limitations and weaknesses. Liquid chromatography-mass spectrometry (LC–MS) is needed to study complex mixtures. In vivo studies are necessary to complete in vitro data if plant extracts exhibit possible biological effects against pathogenic bacteria and anti-proliferative activities against cell lines. In the in vivo context, target organ delivery, efficacy, and possible adverse effects are far more complicated. Additional research is required to determine, which AARE molecules are beneficial and which are harmful, as well as their potential for biological activity. Our group intends to go a step further and employ animal models to preclinically investigate the bioactivities of the AARE.

## 4. Materials and Methods

### 4.1. Plant Material and Preparation

The air-dried root of *A. absinthium* was sourced from a local market in Riyadh, Saudi Arabia. The Department of Botany and Microbiology, College of Science, King Saud University, Riyadh, Saudi Arabia (KSU NO-17795) herbarium has voucher specimens of the plant species that were chosen after validation by Prof. Dr. Mohammed. To remove surface contaminants, the roots were rinsed thoroughly with running water, then left to cure at room temperature for ten days. As previously mentioned [66], the dried root was ground into a fine powder using a tissue crusher. For chemical analysis, 100 mg of the powdered sample was mixed with 2 mL of 70% methanol and sonicated for 30 min at 25 °C and 40 KHz. The mixture was then filtered through a 0.2 μm membrane and centrifuged at 13,000× *g* rpm for 10 min, and the supernatant was used for the analysis. The resulting solution was concentrated to 50 mg/mL (*w*/*v*) for further analysis. Using the same extraction procedure as previously mentioned, another 100 mg of the sample powder was extracted to yield 2 L of extract, which was then allowed to settle and filtered. To assess pharmacological activity, the extract was filtered, dried in a freeze-dry dryer, and condensed into a paste using a rotary evaporator.

### 4.2. Identification of Bioactive Compounds

Bioactive components of a GC-MS system were identified, in conjunction with an Agilent 5977A MSD system that included a DB5-MS column (30 m length × 0.25 mm internal diameter, phase thickness 0.25 μm) from Agilent Technologies (Santa Clara, CA, USA) [82]. A steady temperature of 7.5 °C per minute was intended, with intervals of 40 °C for three minutes, 280 °C for five minutes, and 290 °C for one minute. The GC-MS was used to gather the data at an ionization voltage of 70 eV and an ionization mode of EI. The injector and detector were calibrated to operate at 200 °C and 300 °C, respectively. The MS specs were for a consistent flow rate of 1 mL/min for the carrier gas (helium), and a quantity scanning range of 20 to 500 amu. The Willey and the National Institute of Standards and Technology (NIST) Mass Spectral Data Base served as the search libraries. The parts with a matching factor of more than 90% were discovered by comparing the components with those in the computer NIST libraries linked to the GC-MS equipment.

### 4.3. Determination of TPC and TFC

TPC was measured using the Folin–Ciocalteu method as described in [83], with the gallic acid standard (10–100 mg/mL). Following the appropriate concentration of the extract, 0.25 mL of extract was mixed with 1.25 mL of the Folin–Ciocalteu reagent (diluted five times with distilled water) and 7.5% NaHCO_3_. This mixture was allowed to settle at 45 °C for 15 min, and then the absorbance at 765 nm was measured using a spectrophotometer (U2001 UV–vis Spectrophotometer, Hitachi, Japan). Using the coefficient of determination (R^2^) of the calibration curves, the linearity of the curve was evaluated. Gallic acid equivalent (mg GAE)/g of extract was used to express the findings.

The TFC was measured using aluminum chloride (AlCl_3_) colorimetric method content in the crude extract reported by [84]. The extract was mixed with 0.5 mL of methanol, 20 mg/mL of extract, and 0.5 mL of extract. Following that, 0.1 mL of 10% AlCl_3_ and 1.0 M sodium acetate were added to the samples. After 2.8 mL of filtered water was added, the solutions were left to settle at room temperature. The absorbance of the reaction solutions at 415 nm after 30 min was measured using the Hitachi U2001 UV–vis Spectrophotometer (U2001 UV–vis Spectrophotometer, Hitachi, Japan). The same method was used to produce the calibration curve for quercetin as standard solutions (10–100 mg/mL). Vitamin C was used as the positive control. The results were expressed as milligrams of gallic acid equivalents (mg QE/)/g of extract.

### 4.4. Antioxidant Activity

#### 4.4.1. DPPH Assay

DPPH radical scavenging activity was assessed [85], and an ascorbic acid (200 μL, 50–800 μg/mL) was used as the positive control. In brief, the methanol extract (0.2 mL) was mixed with 2 mL of a 0.08 mM DPPH solution, and the combination was then left to incubate for 20 min in the dark. Using a spectrophotometer (U2001 UV–vis Spectrophotometer, Hitachi, Japan), the mixture’s absorbance at 517 nm was measured after incubation. IC_50_ values were calculated using Graph Pad Prism software (version 5.0, La Jolla, CA, USA).

#### 4.4.2. ABTS Assay

The ABTS radical scavenging assay was performed following the methodology outlined by [86]. The ABTS solution (192 mg/50 mL) and the potassium per-sulfate solution (140 mM) were first combined to perform the test. As a result, the reaction mixture was left at room temperature in the dark for around twelve hours. Moreover, methanol was added to the ABTS solution to provide an absorbance of 0.70 ± 0.02 at 734 nm. Additionally, 3 mL of diluted ABTS were thoroughly combined with 50 µL of each concentration of AARE. Additionally, the mixture was incubated in a dark environment for six minutes. After that, a spectrophotometer (U2001 UV–vis Spectrophotometer, Hitachi, Japan) was used to measure the absorbance at 734 nm. It should be noted that ascorbic acid (µg/mL) was used as the positive control in this investigation. The results were expressed as ABTS% inhibition and IC_50_ values.

### 4.5. Cell Culture and Cytotoxicity Assays

The Hepatocellular carcinoma cell (HepG2) (ATCC HB-8065) and Michigan Cancer Foundation-7 (MCF-7) (ATCC HTB-22) are the most commonly used cancer cell lines for evaluating the plant extract’s cytotoxicity. MCF-7 breast cancer adenocarcinoma cells, derived in 1970, are the most widely used breast cancer cell line in the world. HepG2 was patented in 1980 by researchers at the Wistar Institute in Philadelphia [87,88].

The cells were cultured in Dulbecco’s Modified Eagle Medium (DMEM), which was supplemented with fetal calf serum (FCS) and 1% penicillin-streptomycin. The cells were cultivated in a humidified atmosphere with 5% CO_2_ at 37 °C. Extract concentrations ranging from 50 to 400 µg/mL were prepared in DMEM with 0.1% dimethyl sulfoxide (DMSO). Next, the extract-treated cells were incubated at 37 °C and 5% CO_2_ for 24 h. The cisplatin (30 µg/mL) was used as a positive control, while the negative control was the untreated cells. The MTT solution (10 µL, 5 mg/mL) was added to each well. The plates were then incubated at 37 °C for 4 h at 37 °C and 5% CO_2_. The plates were then incubated for four hours at 37 °C using the previously specified conditions. The wells were then filled with 100 μL of DMSO and incubated for ten more minutes. A microtiter plate reader (BioTek Laboratories, LL, Shoreline, WA, USA) equipped with an ELX-808 microplate reader was used to measure the samples’ absorbance at a wavelength of 590 nm. The cell viability (%) was then calculated. Notably, the experiment was performed in triplicate to ensure reliable results. The mean value was considered for data analysis, and the IC_50_ value was subsequently determined using the GraphPad Prism software (version 5.0, La Jolla, CA, USA).

### 4.6. RT-qPCR Assay of Apoptosis Genes

Gene expression of apoptotic (*caspase-3*, -*8*, -*9*, *Bax*) and anti-apoptotic (*Bcl-xL*, *Bcl-2*) markers was determined via RT-PCR using RNA extracted from HepG2 and MCF-7 cells treated with 150 µg/mL of the plant extract. The cell pellets from samples after centrifugation at 3000× *g* rpm for 5 min at 4 °C were used for RNA extraction using an RNeasy kit (Qiagen, Hilden, Germany). RT-qPCR methodology was conducted as outlined in our recent work using a one-step RT^2^ SYBR^®^ Green/ROX™ qPCR Master Mix [89]. For each sample, a master mix was prepared by combining the following components: Go Taq Qpcr master mix (2×) (10 µL), one-step RT mix (0.4 µL), CXL (0.33 µL), F primer (0.4 µL), R primer (0.4 µL), RNase-free water (8.5 µL), and RNA template (4 µL). The 7500 Fast real-time PCR (7500 Fast; Applied Biosystems, Waltham, MA, USA) was used to perform the RT-qPCR reactions under the following process conditions: initial PCR denaturation at 95 °C for 10 min and reverse transcription at 37 °C for 15 min. Denaturation at 95 °C for 10 s, annealing at 60 °C for 30 s, and extension at 72 °C for 30 s comprised each PCR cycle. Forty cycles in all were completed. To make sure there were no primer dimers present and to validate the specificity of the amplification, a melting curve analysis was carried out. The results were analyzed using the 2^−ΔΔCq^ method, as described [90]. Additionally, the expression of GAPDH amplified from the same samples was used to normalize the Delta Cq (^ΔCq^) values that were obtained for the various genes. The expression of the untreated control cells was then compared to determine the fold change in expression. Table 5 lists the particular oligo sequences that were used for the PCR amplification.

### 4.7. Antidiabetic Activity

#### In Vitro α-Amylase and α-Glucosidase Inhibition Assay

The inhibition of α-amylase was determined using the 3,5-dinitrosalicylic acid (DNSA) method [95]. Using the buffer (0.02 M Na_2_HPO_4_/NaH_2_PO_4_; 0.006 M NaCl; pH 6.9), AARE was diluted to achieve concentrations ranging from 50 to 1000 μg/mL. After mixing 200 µL of the extract with 200 µL of the Molychem α-amylase solution (2 units/mL), the mixture was incubated for 10 min at 30 °C. After that, 200 µL of the 1% starch solution (*w*/*v*) was added to each tube, and they were left for three minutes. In total, 200 µL of DNSA reagent (12 g of sodium potassium tartrate tetrahydrate in 8.0 mL of 2 M NaOH and 20 mL of 96 mM 3,5-DNSA solution was added to stop the reaction, and it was then heated for 10 min at 85 °C in a water bath. Acarbose (Bayer) (100 μL of 25 to 400 µg/mL) was used as a positive control. A UV–visible spectrophotometer (U2001 UV–vis Spectrophotometer, Hitachi, Japan) was used to measure the absorbance at 540 nm after the sample had cooled to room temperature and been diluted with 5 mL of distilled water.

α-glucosidase inhibition was assessed using the p-nitrophenyl-α-D-glucopyranoside (pNPG) method [95]. To achieve a final concentration of 0.5 to 5.0 mg/mL, AARE or Acarbose (a positive control) (100 μL of 2 to 20 mg/mL) was mixed with 50 μL of α-glucosidase (1 U/mL) generated in 0.1 M phosphate buffer (pH 6.9) and 250 μL of 0.1 M phosphate buffer. At 37 °C, the mixture was pre-incubated for 20 min. After pre-incubation, 10 μL of 10 mM pNPG prepared in a 0.1 M phosphate buffer (pH 6.9) was added, and the mixture was incubated at 37 °C for 1 h. The absorbance was measured at 405 nm using a UV–vis spectrophotometer (U2001 UV–vis Spectrophotometer, Hitachi, Japan) after the reactions were stopped by adding 650 μL of 1 M sodium carbonate. The results were represented as a percentage of the enzyme activity inhibition (%), and IC_50_ values were calculated using GraphPad Prism.

### 4.8. Antibacterial Activity

#### 4.8.1. Disk Diffusion Assay

The disk diffusion technique was used to determine the antibacterial activity of AARE against Gram-positive bacteria (*S. aureus*, ATCC -23235; *S. epidermidis,* ATCC-12228; *B. subtilis*, ATCC- 23857) and the Gram-negative bacteria (*E. coli*, ATCC-25922; *P. aeruginosa*, ATCC-27853; *K. pneumonia*, ATCC-13883), as previously mentioned [96]. The negative control was Muller Hinton Broth (HMB) with 0.1% DMSO, whereas the positive control was chloramphenicol (25 µg/mL). Briefly, a Whatman No. 1 filter paper (6 mm diameter) was coated with 20 µg AARE. Using a “L” rod, 0.1 mL of bacterial inoculums were spread to MHA plates. Muller Hinton Agar (MHA) plates were used to hold the sample and standard disk, and they were incubated for twenty-four hours. The antibacterial efficiency was assessed by measuring the zone of inhibition in (mm) around each plant extract-containing well against the microorganisms under investigation.

#### 4.8.2. MIC and MBC

The MIC and MBC of AARE were determined using the broth dilution technique [97] using the 2,3,5-triphenyl tetrazolium chloride (TTC) method, as mentioned in our recent work [98]. MHB (100 μL) was combined with varying amounts of AARE (1.95 to 800 μg/mL) on a 96-well plate. Following the addition of 10 µL of bacterial solution, each well had a total bacterium count of 5 × 10^6^ colony-forming unit (CFU)/mL. The positive control included 25 μg/mL of chloramphenicol, whereas the negative control contained 0.1% DMSO with HMB. Following a 24 h incubation period at 37 °C, the plates were visually inspected for the presence of bacteria. The mixture was then incubated for 20 min at 37 °C after adding 2 mg/mL of TTC working solution in PBS to each well. Pink solution wells that resembled the positive control were regarded as positive for bacterial growth, while colorless solution wells were regarded as negative. The MIC was recognized as the lowest concentration preventing bacterial growth, and MBC, the lowest concentration showing subculture [99].

### 4.9. Molecular Docking

Molecular docking was employed to theoretically assess the potential antioxidant, antibacterial, and anticancer effects of the AARE.

#### 4.9.1. Ligand Preparation

The ligand preparation process started with retrieving the compounds in AARE from the PubChem Data Base in Structure-Data file (SDF) format. The LigPrep module of Schrödinger software (version 11.5) was utilized to refine these ligands using the Optimized Potentials for Liquid Simulations3 (OPLS3) force field. Each molecule was optimized for its ionization state at a pH of 7.0 ± 2.0, allowing for generating up to 32 potential stereoisomers per compound [100,101].

#### 4.9.2. Protein Preparation

The proteins selected for the docking study were obtained from the Protein Data Bank, specifically the structures of human NADPH oxidase (PDB ID: 2CDU), *E. coli* gyrase B (PDB ID: 3G7E), and topoisomerase IIα (PDB ID: 1ZXM). The preparation of these proteins involved structural refinement, including the addition of hydrogen atoms, correction of bond orders, removal of water molecules, assignment of hydrogen bonds, optimization of receptor atom charges, and energy minimization using the OPLS3 force field [102,103].

#### 4.9.3. Glide Standard Precision (SP) Ligand Docking

Flexible ligand docking was carried out using the Standard Precision (SP) mode in Glide, part of Schrödinger-Maestro version 11.5. Non-cis/trans amide bonds were penalized during the docking procedure. Van der Waals interactions for ligand atoms were scaled at 0.80, while the partial charge cutoff was fixed at 0.15. The docking outcomes were assessed using the glide score, calculated from the energy-minimized ligand poses. The pose with the lowest glide score for each ligand was chosen as the optimal docking result [104,105].

### 4.10. ADMET Analysis

ADMET parameters were predicted using the QikProp module within Schrödinger’s Maestro software (version 11.5). The predictions were based on the physicochemical and pharmacokinetic properties of the molecules analyzed in this study, including molecular weight, hydrogen bond donors and acceptors, total solvent-accessible surface area, bloodؘ–brain partition coefficient, octanol/water partition coefficient, and aqueous solubility [106].

### 4.11. Statistical Analysis

All of the data gathered about the efficacy of various extract concentrations were analyzed using one-way analysis of variance (ANOVA), with a post hoc least significant difference test (*p* < 0.05). All experiments were conducted in triplicate.

## 5. Conclusions

The methanolic extraction of AARE yielded a wide range of bioactive chemicals, with 5-hydroxymethylfurfural being the most abundant. Other prominent chemicals, such as 4H-Pyran-4-one and 2,3-dihydro-3,5-dihydroxy-6-methyl, show their potential for antioxidant and antibacterial therapy, which is consistent with the plant’s traditional applications. AARE had a greater TPC than TFC, confirming its antioxidant ability, as indicated by moderate DPPH and ABTS radical scavenging activity. The extract also showed strong anticancer action against MCF-7 and HepG2 cells, inducing apoptosis by upregulating pro-apoptotic genes while downregulating anti-apoptotic markers. AARE inhibits α-amylase and α-glucosidase enzymes, which cause postprandial hyperglycemia, indicating potential antidiabetic benefits. Its inhibitory efficacy was equivalent to that of acarbose, suggesting that it might be used as a natural antidiabetic drug. AARE had high antibacterial activity, particularly against Gram-positive bacteria, making it a potential option for natural antibacterial therapy. Molecular docking investigations found compounds with potential antioxidant, antibacterial, and anticancer properties, including 2,4-Dihydroxy-2,5-dimethyl-3(2H)-furan-3-one (NADPH oxidase inhibitor), 1H-Purin-2-amine (*E. coli* Gyrase B inhibitor), and 4H-Pyran-4-one (Topoisomerase IIα inhibitor). Future studies also should include in vivo investigations that investigate the molecular processes behind AARE’s bioactivity and possible synergistic effects, as well as clinical trials to validate its therapeutic effectiveness in cancer, diabetes, and bacterial infections.

## Figures and Tables

**Table 1:** GC-MS compounds in AARE.

Peak	RT	Area	Area%	Name	MF	MW	Classification
1	4.416	216,109,638	2.48	2-Furanmethanol	C5H6O2	98	Heteroaromatic compounds
2	5.1	39,307,282	0.45	4-Cyclopentene-1,3-dione	C5H4O2	96	Beta-diketones
3	6.643	1,153,012,321	13.26	1,5-Hexadien-3-ol	C6H10O	98	Secondary alcohols
4	7.005	199,434,450	2.29	2,4-Dihydroxy-2,5-dimethyl-3(2H)-furan-3-one	C6H8O4	144	Furanones
5	7.555	133,154,934	1.53	N-Methyl-3-hydroxymethylpyrrolidin-2-one	C6H11NO2	129	N-alkylpyrrolidines
6	8.05	59,776,143	0.68	Alpha-l-rhamnopyranose	C6H12O5	146	Monosaccharides
7	9.712	191,174,820	2.19	Furaneol	C6H8O3	128	Furanones
8	9.87	297,136,547	3.41	2,5-Dimethylfuran-3,4(2H,5H)-dione	C6H8O3	128	Furanones
9	12.032	1,248,467,915	14.35	4H-Pyran-4-one, 2,3-dihydro-3,5-dihydroxy-6-methyl-	C6H8O4	144	Dihydropyranones
10	13.163	161,755,285	1.86	4H-Pyran-4-one, 5-hydroxy-2-(hydroxymethyl)-	C6H6O4	142	Pyranones and derivatives
11	13.841	79,467,628	0.91	5-Acetoxymethyl-2-furaldehyde	C8H8O4	168	Aldehydes
12	15.243	450,3121,608	51.79	5-Hydroxymethylfurfural	C6H6O3	126	Aldehydes
13	17.155	62,389,788	0.71	Cyclohexanecarboxylic acid, 2-hydroxy-, ethyl ester	C9H16O3	172	Secondary alcohols
14	20.342	4,955,677	0.05	4-Pyridinecarboxylic acid, 3-hydroxy-5-(hydroxymethyl)-2-methyl	C8H9NO4	183	Pyridinecarboxylic acids
15	24.335	13,248,571	0.15	1H-Purin-2-amine, 6-methoxy-N-methyl-	C7H9N5O	179	Purinones
16	29.733	5,391,479	0.06	Palmitoleic acid	C16H30O2	254	Long-chain fatty acids
17	30.159	8,772,084	0.101	Hexadecanoic acid, methyl ester	C17H34O2	270	Fatty acid methyl esters
18	30.52	21,018,337	0.24	9-Hexadecenoic acid	C16H30O2	254	Long-chain fatty acids
19	30.976	65,502,216	0.75	n-Hexadecanoic acid	C16H32O2	256	Long-chain fatty acids
20	31.542	17,036,529	0.19	Azuleno [4,5-b]furan-2(3H)-one, decahydro-3,6,9-tris(methylene)-, [	C15H18O2	230	Sesquiterpene lactones
21	33.486	25,977,080	0.29	9,12,15-Octadecatrienoic acid, methyl ester, (Z,Z,Z)-	C19H32O2	292	Lineolic acids and derivatives
22	33.577	13,489,678	0.15	9-Octadecenoic acid (Z)-, methyl ester	C19H36O2	296	Fatty acid methyl esters
23	34.308	138,676,983	1.59	9,12-Octadecadienoic acid (Z,Z)-	C18H32O2	280	Lineolic acids and derivatives
24	34.633	9,381,531	0.11	Oleic Acid	C18H34O2	282	Long-chain fatty acids
25	34.769	14,864,213	0.17	cis-13-Eicosenoic acid	C20H38O2	310	Long-chain fatty acids
26	40.327	12,030,466	0.13	Hexadecanoic acid, 2-hydroxy-1-(hydroxymethyl)ethyl ester	C19H38O4	330	Monoacylglycerols

**Table 2:** The inhibitory zone (mm), MIC (μg/mL), and MBC (μg/mL) of ROLEO.

Bacterium/ Dilution	Positive Control	400 μg/mL	200 μg/mL	100 μg/mL	50 μg/mL	MIC (μg/mL)	MBC (μg/mL)
*Staphylococcus aureus*	24.33 ± 2.08	19.00 ± 2.11	16.00 ± 2.21	12.33 ± 1.42	9.00 ± 2.16	3.12 ± 0.00	6.25 ± 0.00
*S. epidermidis*	21.00 ± 2.31	19.33 ± 1.21	16.22 ± 2.84	14.38 ± 2.36	10.23 ± 1.55	3.12 ± 0.00	6.25 ± 0.00
*Bacillus subtilis*	23.66 ± 2.42	18.00 ± 1.35	14.00 ± 1.36	12.12 ± 1.66	9.23 ± 2.11	6.25 ± 0.00	12.50 ± 0.00
*Escherichia coli*	21.22 ± 1.12	16.23 ± 2.61	12.00 ± 1.57	11.00 ± 1.33	7.00 ± 0.29	25.00 ± 0.00	50.00 ± 0.00
*Klebsiella pneumonia*	25.00 ± 1.13	13.22 ± 1.36	10.21 ± 1.32	8.22 ±1.96	6.00 ± 1.73	12.50 ± 0.00	25.00 ± 0.00
*Pseudomonas aeruginosa*	24.33 ± 2.18	14 ± 1.38	11.36 ± 1.96	8.33 ± 1.14	7.42 ± 1.36	25.00 ± 0.00	50.00 ± 0.00

**Table 3:** Docking results in ligands in different receptors.

	Glide Gscore (kcal/mol)		
	NADPH (PDB:2CDU)	*E. coli* Gyrase B (PDB:3G7E)	Topoisomerase IIα (PDB: 1ZXM)
9-Octadecenoic acid (Z)-, methyl ester	0.128	−2.291	−1.596
Alpha-l-rhamnopyranose	−4.959	−5.762	−6.681
cis-13-Eicosenoic acid	−3.862	−4.22	−8.526
Hexadecanoic acid, methyl ester	0.604	−0.702	−0.473
1,5-Hexadien-3-ol	−1.292	−2.108	−1.166
1H-Purin-2-amine, 6-methoxy-N-methyl-	−5.599	−6.929	−7.152
2,4-Dihydroxy-2,5-dimethyl-3(2H)-furan-3-one	−7.118	−6.057	−10.796
2,5-Dimethylfuran-3,4(2H,5H)-dione	−6.461	−5.016	−9.042
2-Furanmethanol	−3.717	−5.663	−5.421
4-Cyclopentene-1,3-dione	−5.828	−5.109	−8.806
4H-Pyran-4-one, 2,3-dihydro-3,5-dihydroxy-6-methyl-	−5.695	−5.051	−11.03
4H-Pyran-4-one, 2,3-dihydro-3,5-dihydroxy-6-methyl-	−4.706	−5.633	−10.899
4H-Pyran-4-one, 5-hydroxy-2-(hydroxymethyl)-	−6.503	−4.963	−9.626
4-Pyridinecarboxylic acid, 3-hydroxy-5-(hydroxymethyl)-2-methyl	−5.825	−5.397	−10.798
5-Acetoxymethyl-2-furaldehyde	−3.846	−5.89	−5.421
5-Hydroxymethylfurfural	−3.999	−4.834	−5.387
9,12,15-Octadecatrienoic acid, methyl ester, (Z,Z,Z)-	0.141	−0.429	−0.722
9,12-Octadecadienoic acid (Z,Z)-	−0.817	−1.156	−6.535
9-Hexadecenoic acid	0.119	−1.278	−6.084
Azuleno [4,5-b]furan-2(3H)-one, decahydro-3,6,9-tris(methylene)-	−4.283	−6.233	−5.65
Cyclohexanecarboxylic acid, 2-hydroxy-, ethyl ester	−4.858	−5.304	−5.331
Furaneol	−6.44	−5.073	−9.042
Hexadecanoic acid, 2-hydroxy-1-(hydroxymethyl)ethyl ester	−2.528	−5.029	−6.692
n-Hexadecanoic acid	−0.006	−0.264	−7.725
N-Methyl-3-hydroxymethylpyrrolidin-2-one	−5.265	−5.112	−5.63
Oleic Acid	−0.665	−0.561	−6.221
Palmitoleic acid	1.12	−0.906	−5.611

**Table 4:** ADME prediction.

Title	mol MW	SASA	donorHB	accptHB	QPlogPo/w	QPPCaco	QPlogBB	%HOA
Hexadecanoic acid, 2-hydroxy-1-(hydroxymethyl)ethyl ester	330.507	753.694	2	4	4.22	1732.552	−0.475	96.66
cis-13-Eicosenoic acid	310.519	787.415	1	2.75	5.296	1384.156	−0.689	100
Oleic Acid	282.465	713.449	1	2.75	4.514	1384.156	−0.62	100
9,12-Octadecadienoic acid (Z,Z)-	280.45	641.478	1	2.75	3.987	1384.156	−0.504	100
9-Octadecenoic acid (Z)-, methyl ester	296.492	726.597	1	3	5.05	5399.811	0.218	100
9,12,15-Octadecatrienoic acid, methyl ester, (Z,Z,Z)-	292.461	653.706	1	3	4.497	5399.811	0.252	100
n-Hexadecanoic acid	256.428	696.398	1	2.75	4.184	1384.156	−0.636	100
9-Hexadecenoic acid	254.412	654.069	1	2.75	3.828	1384.145	−0.577	100
Hexadecanoic acid, methyl ester	270.454	709.545	1	3	4.72	5399.811	0.213	100
Palmitoleic acid	254.412	639.497	1	2.75	3.736	1384.156	−0.55	100
1H-Purin-2-amine, 6-methoxy-N-methyl-	179.181	294.923	3	2	0.218	1593.316	−0.061	72.579
4-Pyridinecarboxylic acid, 3-hydroxy-5-(hydroxymethyl)-2-methyl	183.163	320.41	2	4.75	−0.701	480.193	−0.668	70.831
Cyclohexanecarboxylic acid, 2-hydroxy-, ethyl ester	172.224	291.873	1	4	0.237	2832.799	0.168	90.122
5-Hydroxymethylfurfural	126.112	243.465	1	5	−1.201	551.3	−0.425	68.979
5-Acetoxymethyl-2-furaldehyde	168.149	294.839	0	7	−2.156	597.274	−0.459	64.013
4H-Pyran-4-one, 5-hydroxy-2-(hydroxymethyl)-	142.111	323.612	1	4.75	−0.737	631.421	−0.658	72.752
4H-Pyran-4-one, 2,3-dihydro-3,5-dihydroxy-6-methyl-	144.127	342.706	1	3.75	−0.254	905.191	−0.603	78.38
2,5-Dimethylfuran-3,4(2H,5H)-dione	128.127	361.211	1	5	−0.29	2009.414	−0.188	84.364
Furaneol	128.127	359.422	0	2.75	0.481	1832.64	−0.278	88.166
N-Methyl-3-hydroxymethylpyrrolidin-2-one	129.158	338.121	3	3	0.116	1903.893	−0.123	86.325
1,5-Hexadien-3-ol	98.144	249.629	1	1	0.731	4638.96	0.314	96.849
2,4-Dihydroxy-2,5-dimethyl-3(2H)-furan-3-one	144.127	259.514	1	3.75	−0.333	2074.855	−0.032	84.363
4-Cyclopentene-1,3-dione	96.085	213.072	0	4	−1.407	1401.266	−0.052	75.022
2-Furanmethanol	98.101	268.984	1	3	0.032	3970.245	0.215	91.545

mol MW: Mass of molecules (acceptable range: 500 mol). SASA: Total solvent accessible surface area using a probe with a 1.4 radius (acceptable range: 300–1000radius). donorHB: Donor of hydrogen bonds (acceptable range: ≤5). accptHB: Acceptor of hydrogen bonds (acceptable range: ≤10). QPlogPo/w: Predicted octanol/water partition coefficient (acceptable range: −2–6.5). QPPCaco: Predicted apparent Caco-2 cell permeability in nm/s. Caco-2 cells is a model for the gut–blood barrier (˂25-poor; ˃500-great). QPlogBB: Predicted blood–brain partition coefficient (acceptable range: −3–1.2). %HOA: Predicted human oral absorption on 0 to 100% scale (<25% is poor, and >80% is high).

**Table 5:** The primer sequences of various genes involved in apoptosis and anti-apoptotic genes.

Gene Name	Primers Sequence	Reference
*Caspase-3*	F: 5′-GCTGGATGCCGTCTAGAGTC-3′	[91]
	R: 5′-ATGTGTGGATGATGCTGCCA-3′	
*Caspase-8*	F: 5′-AGAAGAGGGTCATCCTGGGAGA-3′	[92]
	R: 5′-TCAGGACTTCCTTCAAGGCTGC-3′	
*Caspase-9*	F: 5′-ATTGCACAGCACGTTCACAC-3′	[91]
	R: 5′-TATCCCATCCCAGGAAGGCA-3′	
*Bax*	F: 5′-GAGCTAGGGTCAGAGGGTCA-3′	[91]
	R: 5′-CCCCGATTCATCTACCCTGC-3′	
*Bcl-2*	F: 5′-ACCTACCCAGCCTCCGTTAT-3′	[91]
	R: 5′-GAACTGGGGGAGGATTGTGG-3′	
*Bcl-XL*	F: 5′-CAGAGCTTTGAACAGGTAG-3′	[93]
	R: 5′-GCTCTCGGGTGCTGTATTG-3′	
	R: 5′-GGGCGGATTAGGGCTTCC-3′	
GAPDH	F: 5′-CGGAGTCAACGGATTTGGTC -3′	[94]
	R: 5′-AGCCTTCTCCATGGTCGTGA -3′	

## Data Availability

The original contributions presented in the study are included in the article, further inquiries can be directed to the corresponding author.
